# Associations Between 30-Day Mortality, Specialist Nursing, and Daily Physician Ward Rounds in a National Stroke Registry

**DOI:** 10.1161/STROKEAHA.118.021518

**Published:** 2018-08-07

**Authors:** Lizz Paley, Elizabeth Williamson, Benjamin D. Bray, Alex Hoffman, Martin A. James, Anthony G. Rudd

**Affiliations:** 1 From the Clinical Effectiveness and Evaluation Unit, Royal College of Physicians, London, United Kingdom (L.P., A.H.); 2 Department of Medical Statistics, London School of Hygiene and Tropical Medicine, United Kingdom (E.W.); 3 School of Population Health and Environmental Sciences, King’s College London, United Kingdom (B.D.B., A.G.R.); 4 Royal Devon and Exeter NHS Foundation Trust, United Kingdom (M.A.J.).

**Keywords:** adult, hospitals, mortality, nursing, stroke

## Abstract

**Background and Purpose—:**

Well-organized stroke care is associated with better patient outcomes, but the most important organizational factors are unknown.

**Methods—:**

Data were extracted from the Sentinel Stroke National Audit Programme of adults with acute stroke treated in stroke hospitals in England and Wales between April 2013 and March 2015. Multilevel models with random intercepts for hospitals were used to estimate the association of each variable with 30-day mortality to estimate the impact of admission to differently organized hospitals.

**Results—:**

Of the 143 578 patients with acute stroke admitted to 154 hospitals, 14.4% died within 30 days of admission. In adjusted analyses, admission to hospitals with higher ratios of nurses trained in swallow screening was associated with reduced odds of death (*P*=0.004), and admission to hospitals with daily physician ward rounds was associated with 10% lower odds of mortality compared with less-frequent ward rounds (95% CI, 0.82–0.98; *P*=0.013). Number of stroke admissions and overall ratio of registered nurses on duty at weekends were not found to be independently associated with mortality after adjustment for other factors.

**Conclusions—:**

If these associations are causal, an extra 1332 deaths annually in England and Wales could be saved by hospitals providing care associated with a ratio of nurses trained in swallow screening of at least 3 per 10 beds and daily stroke physician ward rounds.

## Abstract 2

Supplemental Digital Content is available in the text.

Stroke is a leading cause of death and adult disability worldwide.^1,2^ During the past 20 years, stroke care provision internationally has undergone enormous change as evidence from controlled trials^3,4^ has demonstrated the effectiveness of interventions (both medical and structural), and many services have reorganized to deliver these aspects of care.^5,6^ The provision of organized stroke unit care benefits all patients, reducing death or dependency at 12 months^3^ and 10 years after stroke.^7^ Therefore, the disability benefit from organizational aspects of care is estimated to be larger than the corresponding benefit from all medical interventions together.^8^

Previous analyses demonstrated associations between the provision of care and outcomes in large stroke registers. For example, stroke specialist physician review within 24 hours of admission, nutrition screening and formal swallow assessment within 72 hours, and antiplatelet therapy, adequate fluid, and nutrition for first 72 hours are associated with lower odds of 30-day mortality.^9^ Which aspects of the organization of stroke unit care are particularly associated with improved outcomes is less clear.

One possibility is that the benefit derives from better prevention and treatment of complications,^10^ through the use of stroke specialist staffing. Key components likely include the timely provision of screening, assessment, and management for common complications, such as urinary tract infections and pneumonia.^11,12^ A single-blind randomized controlled trial demonstrated reduced death and dependency after implementation of evidence-based protocols to manage fever, hyperglycemia, and swallowing dysfunction.^13^

Specific organizational aspects of stroke care have been shown to be associated with outcomes in different countries, such as an association between weekend nurse staffing ratios and mortality in England^14^ and between volume of stroke admissions and reduced mortality in Canada and Japan.^15,16^

However, there is little evidence on the relative importance of factors because previous studies were constrained by the amount of data available, limiting the ability to account for patient characteristics, such as stroke severity,^15^ and the clustered nature of the data,^17^ or did not adjust for in-depth detail on multiple organizational aspects of the stroke care available.^15,16^ We, therefore, set out to use a large, comprehensive national stroke registry to examine the various organizational and patient-level factors that determine early mortality after acute stroke care.

## Methods

Because of the sensitive nature of the data collected for this study, requests to access the data set from qualified researchers trained in human subject confidentiality protocols may be sent to the Healthcare Quality Improvement Partnership at https://www.hqip.org.uk/national-programmes/accessing-ncapop-data/. Patient data were extracted from the national register (Sentinel Stroke National Audit Programme [SSNAP]) of adults (aged ≥16 years) with acute stroke admitted to 100% of acute hospitals in England and Wales from April 2013 to March 2015. The SSNAP data include an estimated 95% of consecutive adult hospital admissions for stroke compared with routine administrative coding data (Hospital Episode Statistics, Patient Episode Database for Wales). The SSNAP data set includes patient characteristics, stroke type, and details of care processes. Data are entered prospectively by the clinical team via a secure online portal, with real-time data validation checks. SSNAP has permission from the National Health Service Health Research Authority under section 251 of the Health and Social Care Act 2006 to collect patient data without prospective consent. Additional ethical permission was not sought.

Hospital data were extracted from the SSNAP Acute Organisational Audit 2014—a web-based survey completed by stroke clinicians at each hospital measuring the structure and organization of stroke services on July 1, 2014, capturing information on nursing and therapy staffing and skills, frequency of physician ward rounds on the stroke unit, and therapy provision (5, 6, or 7 days per week). Hundred percent of hospitals in England and Wales admitting patients with acute stroke submitted information to this survey.

Thirty-day mortality was ascertained within the SSNAP data set for inpatient deaths and through linkage with the Office of National Statistics death registry for patients who died after discharge from hospital.

This analysis explores the association between hospital-level characteristics and 30-day mortality. Variables were chosen for inclusion by review of the literature^18^ and through discussion with clinicians and take into account expert recommendations made in national clinical guidelines.^19^ The selected variables are as follows:

### From the SSNAP Organisational Audit 2014

- Number of acute stroke beds
- The presence of each type of bed on the stroke unit:
  - Beds solely for use in the first 72 hours of stroke (pre–72-hour beds only)
  - Beds that can be used for both the first 72-hour care and post–72-hour care (mixed beds only)
  - A mixture of both types of beds (both pre–72-hour and mixed beds)
- Availability of continuous physiological monitoring
- Number of registered nurses on duty weekends at 10 AM in the stroke unit
- Staffing in the stroke unit includes nurses trained in swallow screening
- Vacancy rate (unfilled stroke physician posts)
- Seven-day working for occupational therapists and physiotherapists
- Frequency of ward rounds by stroke physician(s)
- Protocols or criteria for admission to the stroke unit

Speech and language therapist working was not included because too few hospitals had 7-day working. Swallow screening was defined as “a formal swallow screen (performed by any member of the team). Presence or absence of the gag reflex is not sufficient because it is proven to be of little prognostic value for the ability to swallow.” Although there is no national accreditation for training in swallow screening, local training would usually follow standards laid out in national recommendations and the national competency framework. This would typically include a course to teach the physiology of swallowing alongside standard protocols.

### From the Patient Registry

- Mean annual number of stroke patient admissions (between 2013 and 2015)
- Median time from arrival at hospital to brain scan for patients with stroke (between 2013 and 2015)
- Age
- Sex
- Stroke type
- Time from stroke onset to arrival at hospital
- Time of day of arrival at hospital
- Premorbid cardiovascular comorbidities:
  - Atrial fibrillation
  - Congestive heart failure
  - Hypertension
  - Diabetes mellitus
  - Prior stroke/transient ischemic attack
  - Premorbid modified Rankin Scale score
  - Whether stroke occurred while the patient was already an inpatient in hospital
  - Stroke severity on arrival at hospital

The median brain scanning time per hospital was included as a marker for the effectiveness of hospitals’ acute care protocols.

Stroke severity was measured either using the full National Institutes of Health Stroke Scale (NIHSS) score in the imputed and complete case analyses (scores, 0–42) or using the level of consciousness on arrival (scores, 0–3).

Patients admitted to hospitals treating <100 patients with stroke during the 2-year period were excluded.

## Statistical Analysis

Multilevel logistic regression models with random intercepts for hospitals were fitted. All patient characteristics were included in all models (Table 1). Age and NIHSS were first tested for nonlinearity using the likelihood ratio test and Akaike Information Criterion in a model using only patient characteristics, with age centered at the mean. Subsequently, NIHSS, age, and age squared were entered linearly in all models. Restricted cubic splines with 5 knots were used for continuous organizational characteristics, after centering at the mean.

**Table 1.**
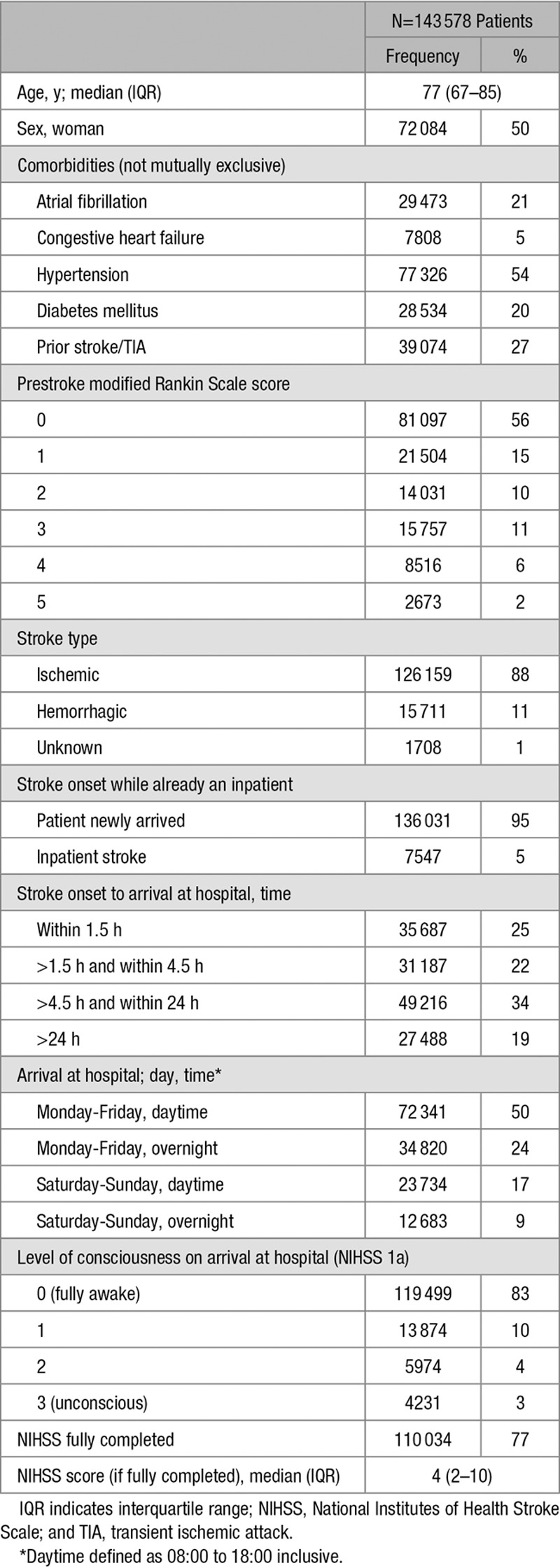
Summary of Patient Characteristics

Minimally adjusted models were first fitted, adjusting for all patient characteristics and only 1 organizational characteristic at a time, using level of consciousness to account for stroke severity. Organizational characteristics were taken forwarded into models adjusting for multiple factors if the likelihood ratio test had *P*<0.25.^20^ The final model was determined by removing variables with *P*>0.05 and rechecking model fit using the likelihood ratio test and the Akaike Information Criterion (Tables 2 and 3).

**Table 2.**
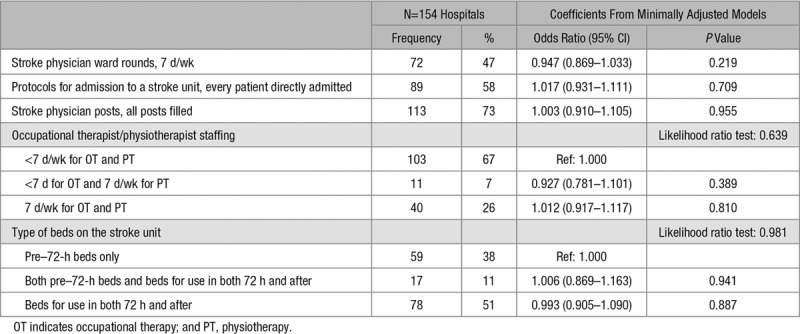
Summary of Hospital Organizational Characteristics, Coefficients From Minimally Adjusted Models (Also Including Patient-Level Characteristics)

**Table 3.**
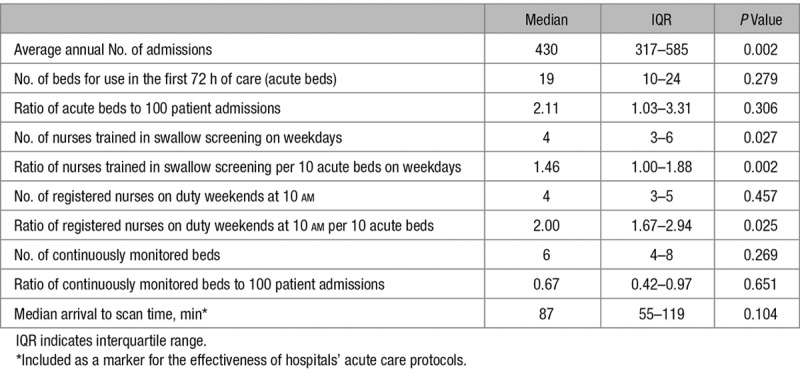
Summary of Continuous Hospital Organizational Characteristics, Coefficients From Minimally Adjusted Models (Also Including Patient-Level Characteristics)

The only variable in the data set with missing data was the full NIHSS. REALCOM-IMPUTE was used to conduct multilevel multiple imputation^21^ with 10 imputed datasets. Full NIHSS was imputed after square root transformation, to reduce skewness. Regression coefficients were combined using Rubin rules.

The organizational characteristics in the final model were the annual number of admissions, the ratio of registered nurses on duty weekends at 10 AM to acute beds, the ratio of nurses trained in swallow screening to acute beds, and 7-day physician ward rounds.

The potential impact on 30-day mortality of changing organizational characteristics was quantified by predicting mortality based on 3 nurses trained in swallow screening per 10 beds for hospitals with lower ratios and 7-day ward rounds for all hospitals. A ratio of 3 nurses per 10 beds was selected from Figure (A) as an illustration of a level associated with lower mortality rates.

**Figure.**
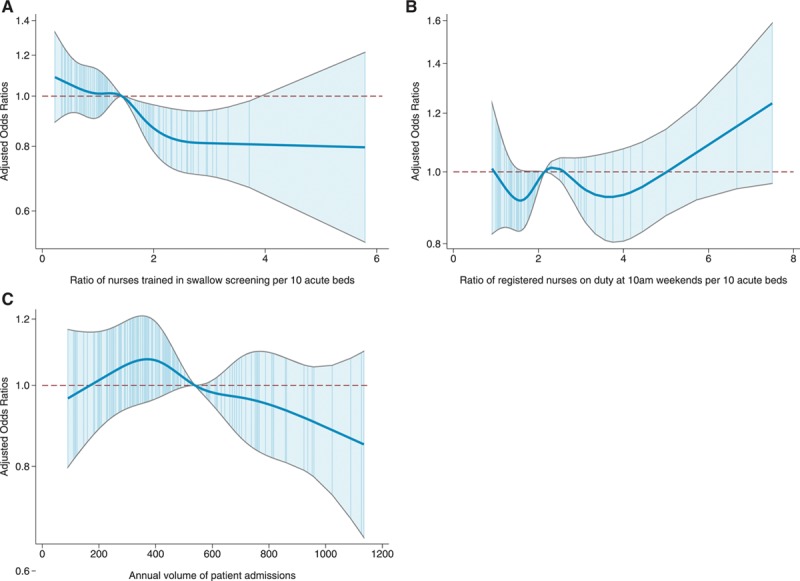
Spline diagrams for hospital-level organizational characteristics in the final model; for the ratio of nurses trained in swallow screening (**A**), the ratio of registered nurses on duty at 10 am weekends (**B**), and the annual volume of patient admissions (**C**). Adjusted odds ratios and 95% CIs where the reference value for each covariate is the median.

Sensitivity analyses were performed using both complete case analysis of only patients with a fully completed NIHSS and a model utilizing a less granular measure of stroke severity available for all patients (level of consciousness on arrival). Three further sensitivity analyses investigated the additional inclusion of the date of treatment, the interactions between imputed NIHSS and daily ward rounds, and ratio of swallow trained nurses per 10 beds.

Statistical analyses were performed using Stata 14.0.

## Results

Data are included on 143 578 patients admitted to 154 hospitals with a total of 2805 stroke unit beds for use in the first 72 hours of care (acute beds) from April 2013 to March 2015. Eighty-eight percent of strokes were ischemic; 83% of patients were fully conscious on arrival at hospital (Table 1); 14.4% of patients died within 30 days of admission to hospital for stroke. Of these deaths, 89.3% were recorded in SSNAP as an inpatient death; the remainder occurred after hospital discharge.

The average annual number of stroke admissions ranged from 90 to 1135 (median, 430; interquartile range, 317–585). The median ratio of all registered nurses on duty weekends at 10 AM was 2.00 nurses per 10 acute beds (interquartile range, 1.67–2.94), whereas the median ratio of nurses trained in swallow screening per 10 acute beds on weekdays was 1.46 (interquartile range, 1.00–1.88; Tables 2 and 3; Figure I in the online-only Data Supplement). There is a weak/moderate positive correlation (*r*=0.38) between the ratio of registered nurses on duty at the weekend and the ratio of nurses trained in swallow screening (Figure II in the online-only Data Supplement).

Crude 30-day mortality varied between hospitals; the median hospital-level 30-day mortality rate was 14.2% (interquartile range, 12.5%–16.3%). There was a strong positive association between age and case fatality and stroke severity and case fatality. For further detail on the association between each variable and mortality, please see Table I in the online-only Data Supplement and Figure III in the online-only Data Supplement.

Table 4 shows the estimated odds ratios for patient characteristics in the final model. After adjustment for factors, including the ratio of all registered nurses on duty at weekends, there remained a significant association between the ratio of nurses trained in swallow screening per 10 acute beds (*P*=0.004) and 30-day mortality. Admission to hospitals with a higher ratio of nurses trained in swallow screening was associated with lower odds of death (Figure [A]). There was no residual association for the ratio of all types of registered nurses (on duty weekends at 10 AM) and mortality (Figure [B]), after adjustment for factors, including the ratio of nurses trained in swallow screening.

**Table 4.**
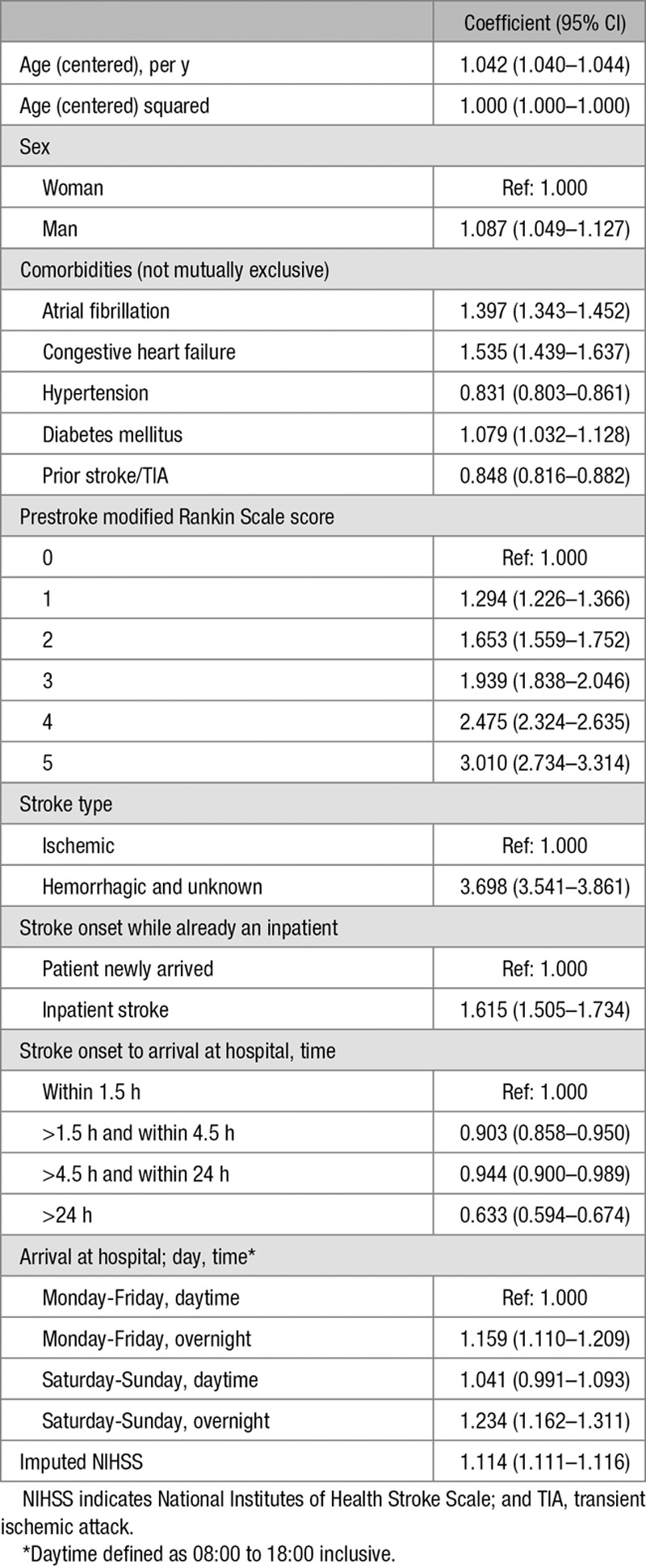
Coefficients for Patient-Level Organizational Characteristics in the Final Imputed NIHSS Model

The adjusted odds of 30-day mortality for a patient admitted to a hospital with 7-day stroke physician ward rounds was 0.90× the odds for a patient admitted to hospital with less-frequent ward rounds (95% CI, 0.82–0.98; *P*=0.013; Table 5).

There was no evidence that the number of admissions was independently associated with 30-day mortality (*P*=0.147; Figure [C]).

To illustrate the potential impact of these findings if they represent a causal relationship; if hospitals with <3 nurses trained in swallow screening per 10 beds increased their ratio to this level and all hospitals had 7-day ward rounds, an estimated 13% of deaths could be avoided, representing an extra 1332 lives saved annually in England and Wales.

Sensitivity analyses were conducted using the NIHSS where fully complete (complete case analysis) and the level of consciousness on arrival instead of the NIHSS. Results for the imputed model differed in important ways from the model using the less granular measure (level of consciousness). In the level-of-consciousness analysis, low-volume hospitals appeared to be associated with the lowest mortality, whereas in the imputed NIHSS model, this was not apparent (Figure [C]), with no overall evidence of an association. Also, there was strong evidence for an association between both the number of admissions and the ratio of registered nurses on duty weekends at 10 AM and 30-day mortality, when the model is adjusted for the level of consciousness of the patient on arrival (Table 6) but not in the imputed NIHSS model (Table 5). The complete case analysis using full NIHSS (Table 6) and the imputed analysis are fairly similar (Table 5), although the independent effect of NIHSS was greater in the complete case analysis than in the imputed analysis.

**Table 5.**
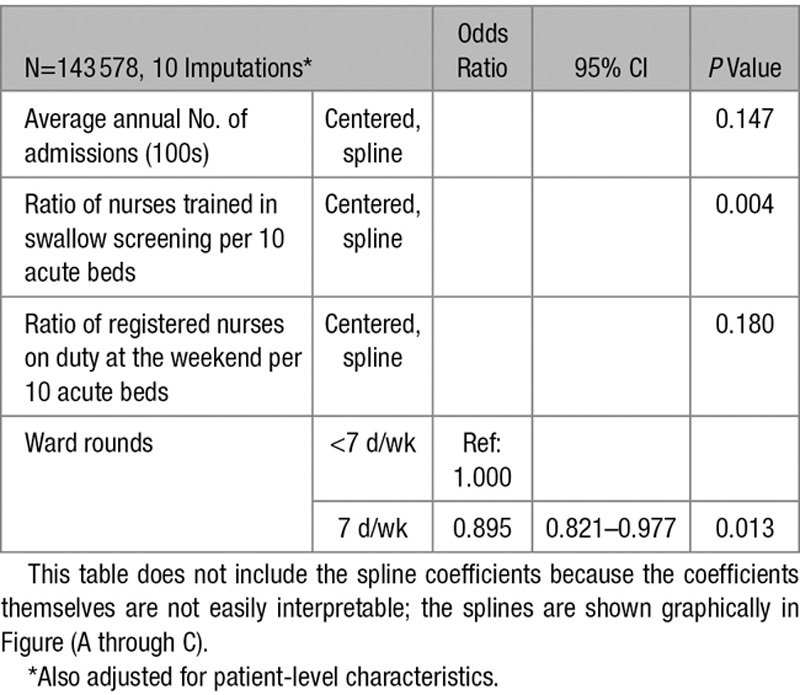
Coefficients for Hospital-Level Organizational Characteristics in the Final Model

**Table 6.**
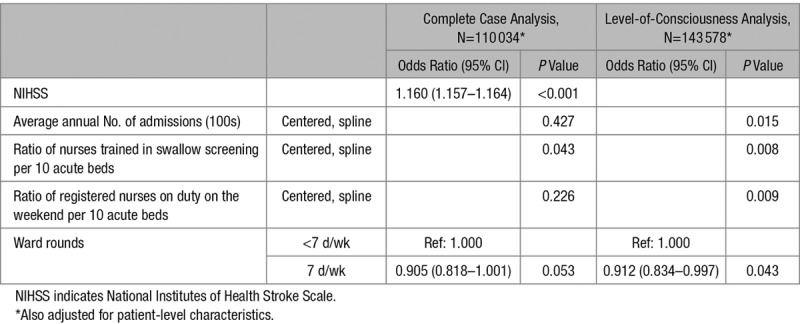
Sensitivity Analyses: Coefficients for Hospital-Level Organizational Characteristics and NIHSS in the Complete Case Model and Level-of-Consciousness Model

There was no evidence that the date of discharge (before or after July 1, 2014) was independently associated with 30-day mortality and little evidence for an interaction between the ratio of swallow trained nurses and NIHSS (Tables II and III in the online-only Data Supplement). However, there was evidence for an interaction between daily ward rounds and NIHSS (adjusted odds ratio, 1.006; 95% CI, 1.002–1.011; Table IV in the online-only Data Supplement). Daily ward rounds were more beneficial for the least severe patients; daily ward rounds for patients with an NIHSS of 0 were associated with a 17% reduction in the odds of death compared with less-frequent ward rounds, whereas there was no difference in the odds of death for patients with an NIHSS of 31. The odds of death associated with daily ward rounds increased for patients with an NIHSS >31, up to a 7% increase for patients with an NIHSS of 42; however, only 1% of patients with stroke have an NIHSS of >31.

## Discussion

Our observational study based on a large, comprehensive national stroke registry found evidence for a reduction in the adjusted odds of death for patients admitted to hospitals with higher ratios of specialist nurses trained in swallow screening and in hospitals with 7-day physician-led ward rounds on the acute stroke unit. The adjusted odds of death plateaued for hospitals with ≥3 nurses trained in swallow screening per 10 acute beds (Figure [A]), suggesting a potential threshold effect for this level of staffing.

After controlling for other factors, there is no evidence that the ratio of all registered nurses on duty at 10 AM weekends or the number of stroke admissions are independently associated with 30-day mortality. Weak/moderate positive correlation between the ratios of the 2 types of nurse staffing suggests considerable variation in the organization of nurse staffing between hospitals.

There was no evidence that other organizational characteristics are associated with 30-day mortality, such as protocols for admission to the stroke unit, the number of continuously monitored beds, and occupational therapy and physiotherapy working at weekends. Almost all hospitals have protocols for direct admission to a stroke unit, except where no stroke unit bed is available; therefore, any survival benefit of protocols for direct admission may already have been conferred on the general UK population, unlike the effects seen for swallow trained nurses and daily ward rounds, which are less commonly available. Alternatively, this lack of an effect may suggest that protocols and beds do not themselves impact mortality, rather the presence of specialist staffing.

The lack of an interaction between NIHSS and the ratio of swallow trained nurses per 10 beds suggests that the association between the ratio of swallow trained nurses and 30-day mortality is consistent across the range of NIHSS.

The evidence for the interaction between NIHSS and daily ward rounds suggests that daily ward rounds seem to have a bigger effect on mortality for less severe patients. However, the interaction’s estimated effect size is small (1.006; 95% CI, 1.002–1.011), and the main effects are largely unchanged. This may be because patients with severe stroke are likely to be seen promptly by stroke consultants regardless of the frequency of ward rounds; therefore, milder patients may benefit the most from increased frequency of ward rounds.

The key finding on the importance of nurses trained in swallow screening is consistent with prior studies demonstrating a reduction in the risk of stroke-associated pneumonia if dysphagia screening was expedited^11^ (dysphagia screening is performed by nurses trained in swallow screening). Stroke-associated pneumonia is a common stroke complication and is associated with a higher risk of death; therefore, interventions that reduce the occurrence of pneumonia could plausibly result in a mortality reduction.

Previous studies have demonstrated the importance of nurse staffing for improved outcomes, and our study might suggest that better management of dysphagia specifically may mediate the link between nurse staffing and reduced mortality.

There are several potential reasons why this analysis did not find an admissions volume effect as documented in previous studies.^15,16^ Higher volume hospitals were more likely to have 7-day ward rounds; therefore, previous studies not adjusting for favorable characteristics, such as staffing, that improve patients’ outcomes would not have elucidated the independent effect of the volume of admissions. This is consistent with the findings from a Taiwanese study showing no independent effect of hospital volume when additionally adjusting for physician volume.^22^ These organizational characteristics are likely to be part of the causal pathway of why higher volume hospitals may produce better outcomes.

Sensitivity analyses demonstrated that when adjusting for the more granular measure of stroke severity (full NIHSS, completed in 77% of cases), other variables were no longer as strongly associated with the outcome than when adjusting only for the level of consciousness on arrival. In particular, the number of admissions was not independently associated with mortality when adjusting using the full NIHSS, yet it was when adjusting only for the level of consciousness, whereas, the interpretation of variables was similar for both analyses using the full NIHSS (complete case analysis and multiple imputation). This suggests that using a more granular measure of stroke severity results in a more nuanced understanding of the interplay between the risk factors, and, therefore, studies that do not use a granular measure of stroke severity may overestimate or underestimate the effect of other variables.

Previous studies have shown that nurse staffing is important for patient outcomes, but these new data imply that hospitals may need to support specialized training, not just ensuring adequate numbers of staff.

The findings of this large, high-quality observational study when taken alongside those of a cluster-randomized clinical trial of protocols to manage swallowing dysfunction^13^ clearly demonstrate that all patients with stroke should now expect to receive swallow screens from individuals trained in the management of swallowing difficulties in patients with stroke.

The main strengths of this analysis are the large data set of unselected patients with stroke, making it representative of real-world stroke care, and that the analysis incorporated information at both patient-level and hospital-level and was able to incorporate detailed information about differences in patients’ age, stroke severity, and the hospitals they were admitted to.

The analysis, however, assumed that organizational factors remained constant throughout the study period, whereas hospitals may have experienced reorganization during the 2-year period, or staff may have left and not been replaced. In addition, although the audit has high levels of case ascertainment (≈95%), and although NIHSS completion rates were relatively high (77%), the rates did vary between centers. This raises the possibility that the missing patients may be a source of unmeasured bias and that NIHSS completion rate in itself may act as a surrogate marker for organizational quality.

This analysis focused on mortality, which is only 1 outcome of stroke, and did not assess reductions in disability or delivery of other potentially important care process measures. This is important because interventions such as thrombolysis do not reduce mortality but do reduce disability. However, although disability at 6 months poststroke is recorded in the audit, only a much smaller subset of patients have this measure recorded, and, therefore, it was not possible to also analyze disability as well as mortality. These differences could also explain previous findings showing that higher volume hospitals are associated with better care processes.^9^

Residual variation was evident between hospitals, even after adjusting for patient- and hospital-level characteristics, suggesting there are potential sources of unmeasured confounding that may act as a source of error and constrain the scope to draw conclusions about causality. For example, the ratio of nurses trained in swallow screening to acute beds is an important association with mortality in this analysis, but it could be that hospitals with a higher ratio of nurses trained in swallow screening also have other unmeasured qualities, which are driving the associations demonstrated here. For example, such hospitals may support more specialized training in general, meaning that other members of the stroke multidisciplinary team are also better trained or nurses trained in swallow screening may have more years of experience than other nurses or be better able to recognize other complications at an earlier stage. Alternatively, speech therapy input could also be an important factor, which was not independently adjusted for in this analysis because of a lack of variation in the speech and language therapy measure included in the organizational audit.

## Conclusions

This analysis demonstrates that key staffing characteristics are associated with the odds of death for patients with stroke and that up to 13% of deaths (1332 patients per year in England and Wales) could potentially be averted if all acute hospitals were able to provide the content of care associated with a ratio of nurses trained in swallow screening of at least 3 per 10 beds and at least daily stroke physician ward rounds.

This analysis did not find evidence for an independent effect of the volume of admissions, and sensitivity analyses suggest that previous findings could be related to incomplete casemix adjustment or to lack of adjustment for other organizational characteristics of stroke units, such as staffing ratios and ward rounds.

## Acknowledgments

We thank the many people and organizations participating in the Sentinel Stroke National Audit Programme (SSNAP), as well as members of the SSNAP collaboration (https://www.strokeaudit.org/Research/SSNAP-Collaboration.aspx).

## Sources of Funding

The Sentinel Stroke National Audit Programme is commissioned by the Healthcare Quality Improvement Partnership and funded by National Health Service (NHS) England and the Welsh Government. No specific funding was sought for this study. Dr James is supported by the National Institute of Health Research (NIHR) Collaboration for Leadership in Applied Health Research and Care for the South West Peninsula. Dr Bray is supported by the NIHR. The views expressed are those of the authors and not necessarily those of the NHS, the NIHR, or the Department of Health.

## Disclosures

None.

## Supplementary Material
