# Supplementary material for: Associations Between 30-Day Mortality, Specialist Nursing, and Daily Physician Ward Rounds in a National Stroke Registry
Source: Stroke. 2018 Aug 7;49(9):2155–62. doi: 10.1161/STROKEAHA.118.021518 (PMC6116797; doi:10.1161/STROKEAHA.118.021518)
Supplement: Supplementary file 1 [file str-49-2155-s001.pdf]

SUPPLEMENTAL MATERIAL

Association between 30-day stroke mortality, specialist acute stroke nursing and daily physician ward rounds in a comprehensive national stroke registry

Supplemental Tables

Table I: 30-day mortality rate for each hospital-level organisational characteristic

		Number of patients	30-day mortality	
		Freq	%	Freq
Overall		N=143,578	14.4%	20,690
Stroke consultant ward rounds:	<7 days a week	60,967	15.4%	9,367
	7 days a week	82,611	13.7%	11,323
Protocols for admission to a stroke unit:	Not every patient directly admitted	54,224	14.5%	7,860
	Every patient directly admitted	89,354	14.4%	12,830
Stroke consultant posts	Some posts unfilled	41,754	14.3%	5,952
	No posts unfilled	101,824	14.5%	14,738
Occupational therapist/ Physiotherapist staffing:	<7 days per week for both OT and PT	87,126	14.7%	12,793
	<7 days for OT and 7 days per week for PT	10,870	13.0%	1,417
	7 days per week for both OT and PT	45,582	14.2%	6,480
Type of beds:	Pre-72h beds only	66,951	13.6%	9,085
	Both pre-72h and mixed beds	17,732	14.3%	2,540
	Mixed beds only	58,895	15.4%	9,065

Table II: Sensitivity analysis including date of discharge

	Odds ratio (95% CI)	p-value
Date of discharge:		
On or before 1st July 2014	Ref: 1.000	
After 1st July 2014	0.998 (0.963 – 1.034)	p=0.910
Daily ward rounds:		
<7-days per week	Ref: 1.000	
7-days per week	0.894 (0.818 – 0.976)	P=0.013
Average annual number of admissions (100s)	*Spline*	P=0.166
Ratio of nurses trained in swallow screening per 10 stroke unit beds	*Spline*	P=0.005
Ratio of registered nurses on duty on the weekend per 10 stroke unit beds	*Spline*	P=0.125

Table III: Sensitivity analysis including interaction between the ratio of swallow trained nurses per 10 acute beds and NIHSS

	Odds ratio (95% CI)	p-value
NIHSS	1.102 (1.083 – 1.122)	P<0.001
Daily ward rounds:		
<7-days per week		
7-days per week	0.894 (0.818 – 0.977)	P<0.013
Interaction between the ratio of swallow trained nurses per 10 acute beds and NIHSS:	*Spline*	P=0.067
Average annual number of admissions (100s)	*Spline*	P=0.158
Ratio of nurses trained in swallow screening per 10 stroke unit beds	*Spline*	P<0.001
Ratio of registered nurses on duty on the weekend per 10 stroke unit beds	*Spline*	P=0.127

Table IV: Sensitivity analysis including interaction between ward rounds and NIHSS

	Odds ratio (95% CI)	p-value
NIHSS	1.111 (1.107 – 1.115)	P<0.001
Daily ward rounds:		
<7-days per week		
7-days per week	0.827 (0.746 – 0.917)	P<0.001
Interaction NIHSS and daily ward rounds:	1.006 (1.002 – 1.011)	P=0.007
Average annual number of admissions (100s)	*Spline*	P=0.161
Ratio of nurses trained in swallow screening per 10 stroke unit beds	*Spline*	P=0.005
Ratio of registered nurses on duty on the weekend per 10 stroke unit beds	*Spline*	P=0.127

Supplemental Figures and Figure Legends

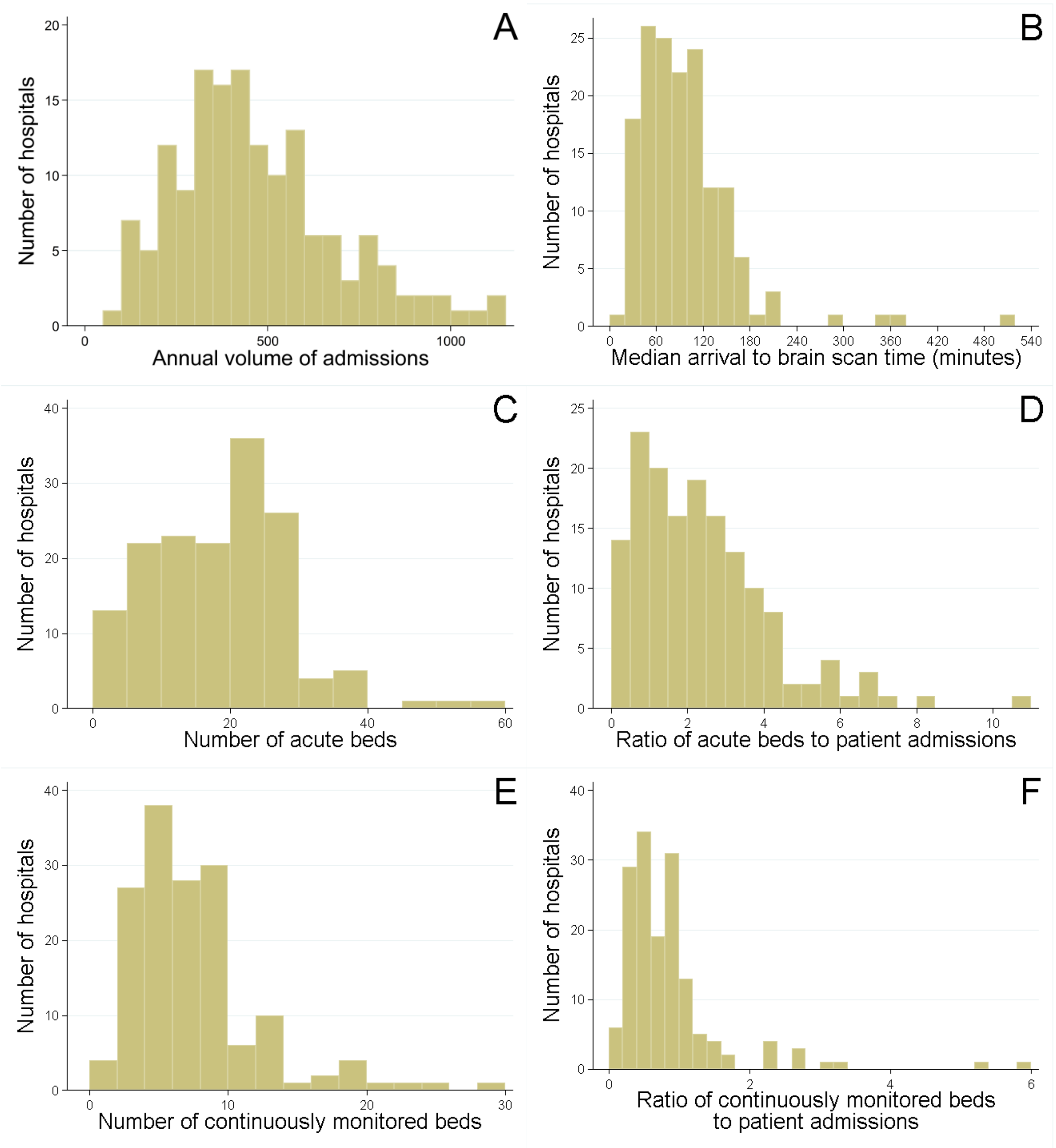

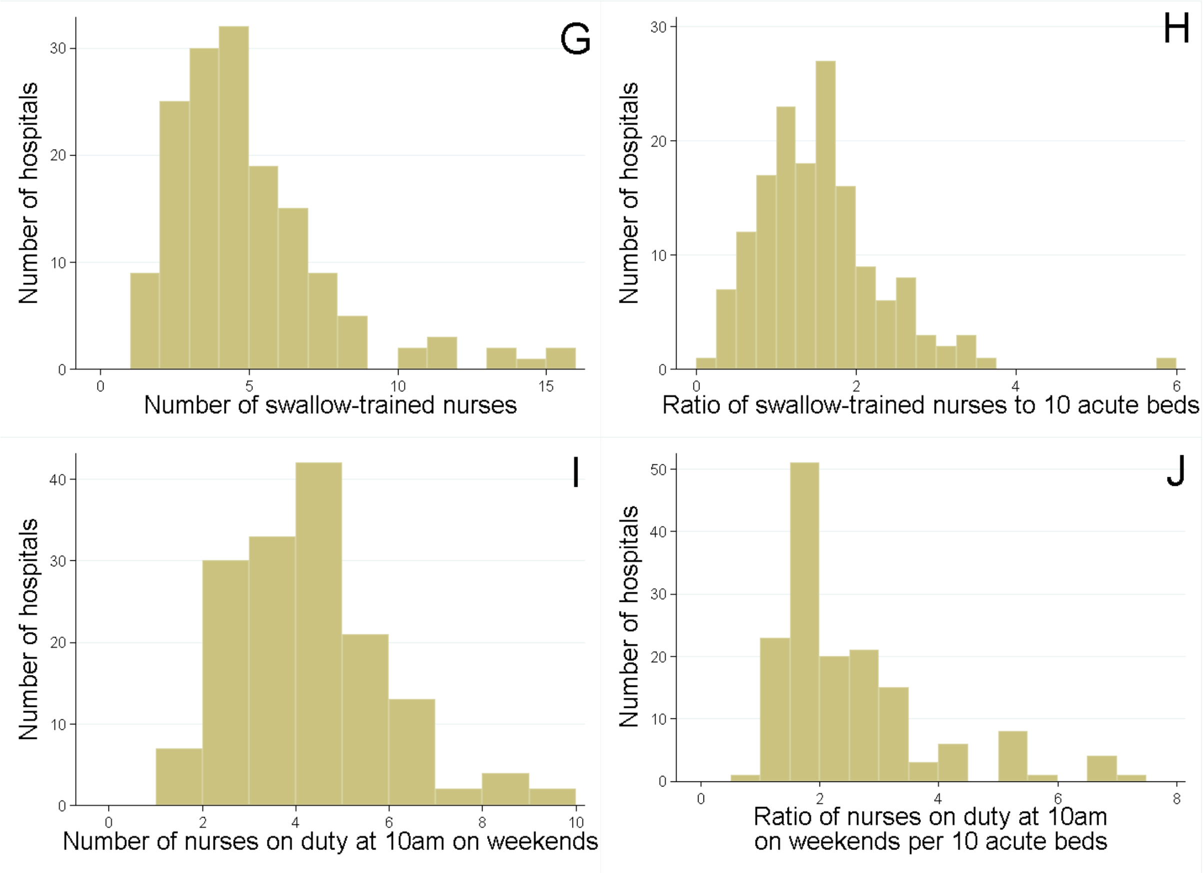

Figure I (A-J): Distributions of hospital-level organisational characteristics

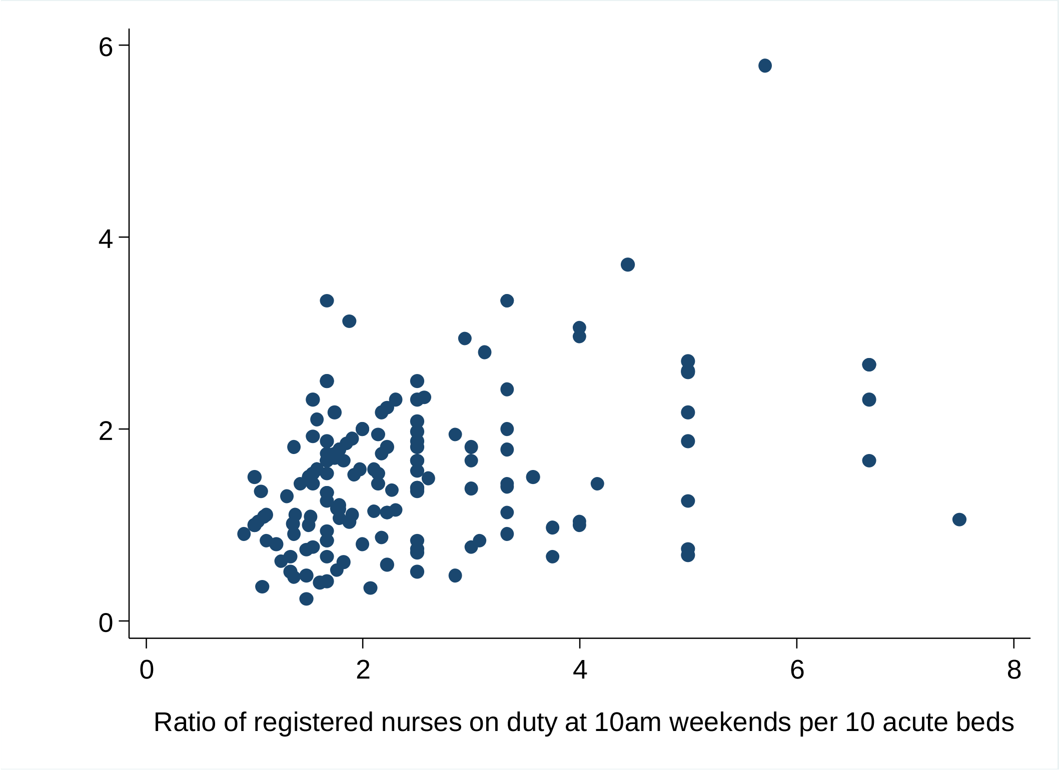

Figure II: Scatter plot for the association between the ratio of registered nurses on duty at 10 am weekends, and the ratio of nurses trained in swallow screening in each hospital

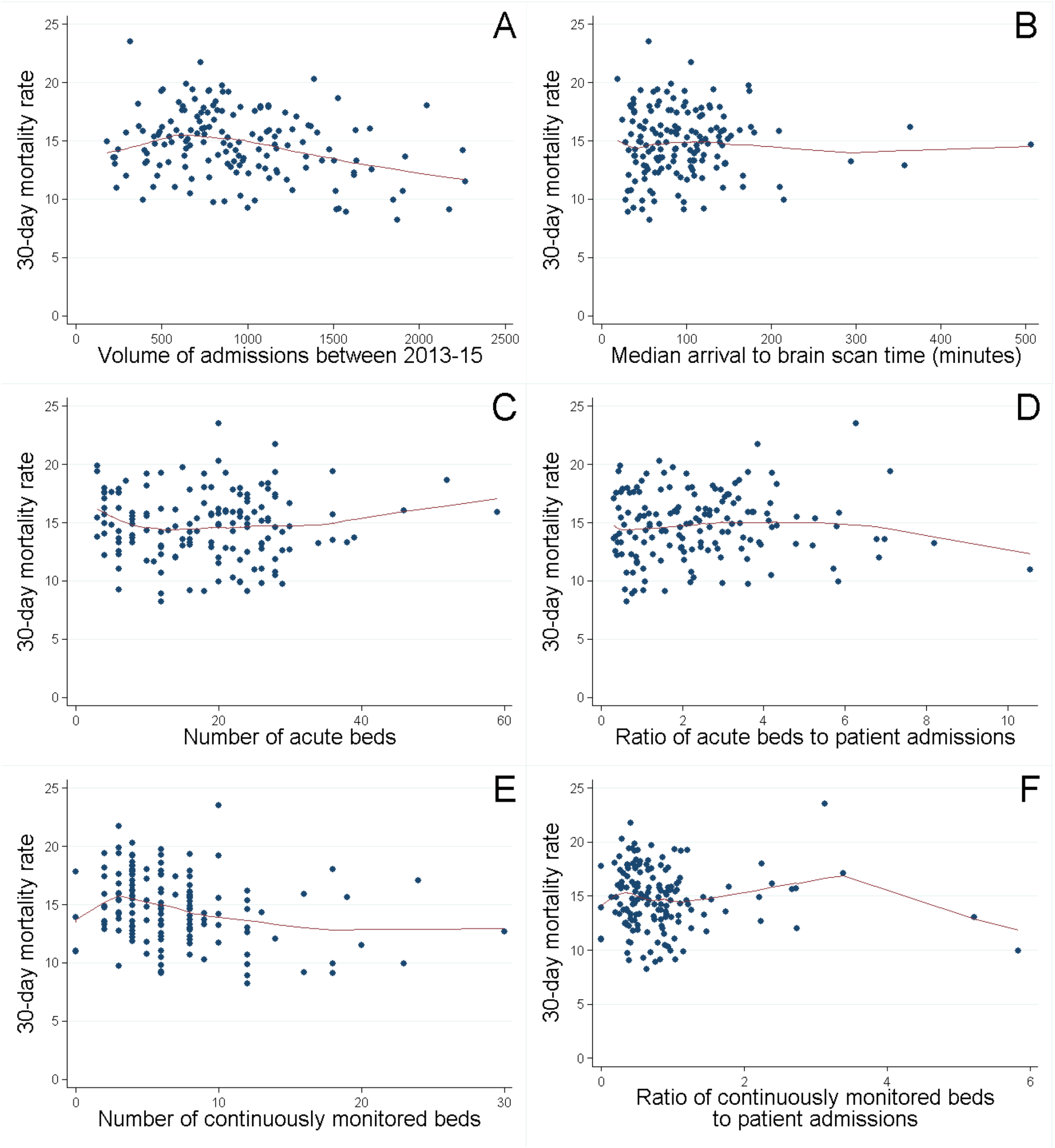
A
B
C
D
E
F
30-day mortality rate
Volume of admissions between 2013-15
Median arrival to brain scan time (minutes)
Number of acute beds
Ratio of acute beds to patient admissions
Number of continuously monitored beds
Ratio of continuously monitored beds
to patient admissions

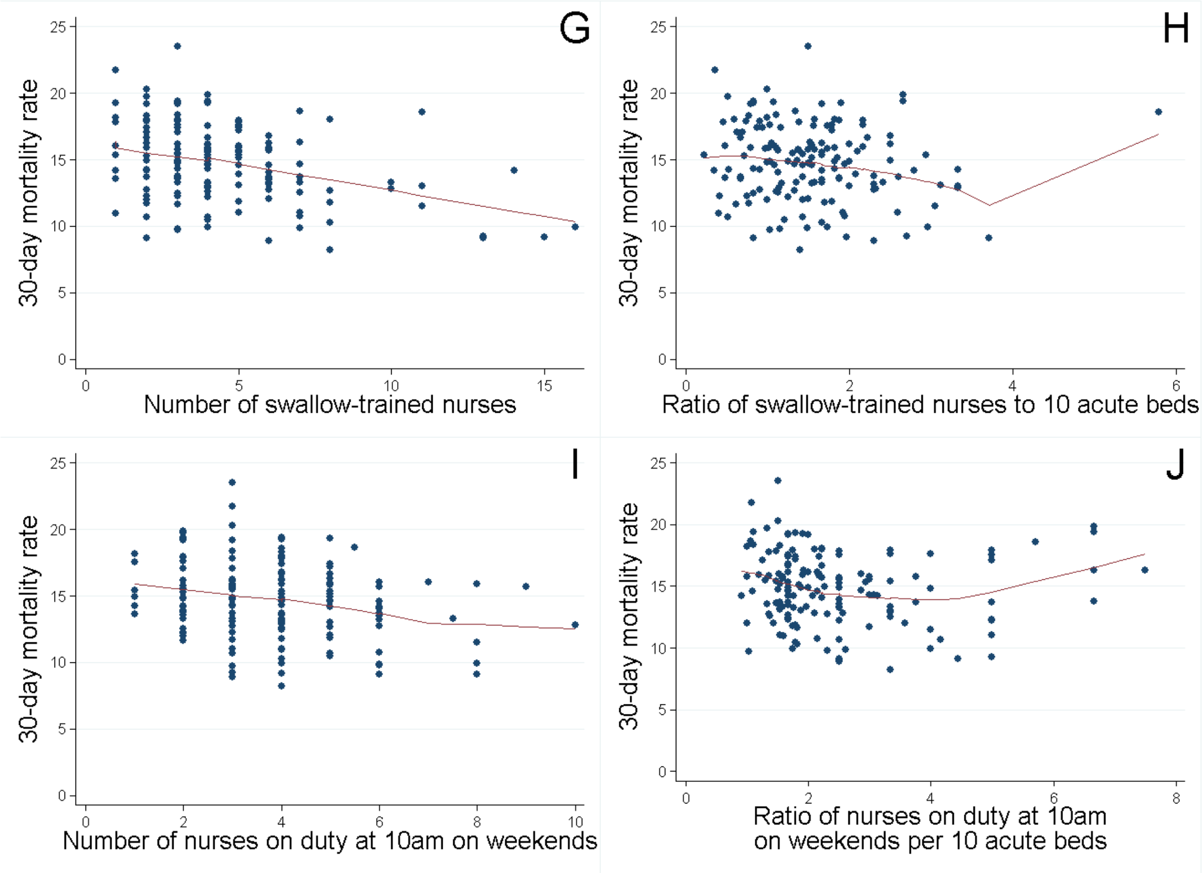

Figure III (A-J): Lowess smoother plots for 30-day mortality rate per hospital for each hospital-level organisational characteristic
